# Long-Term Suitability of Left Gastric Artery Inflow for Arterial Perfusion of Living Donor Right Lobe Grafts

**DOI:** 10.1155/2022/9421648

**Published:** 2022-12-01

**Authors:** Judyta A. Lipinska, Johnny Wang, Joseph N. Carey, Aaron A. Ahearn, Yuri S. Genyk

**Affiliations:** ^1^Department of Surgery, Division of Abdominal Organ Transplant, Hepatobiliary and Pancreas Surgery, University of Southern California, Keck School of Medicine, 1510 San Pablo Street, Building 4300, Suite #412, Los Angeles, CA 90033, USA; ^2^Department of Surgery, Division of Plastic and Reconstructive Surgery, University of Southern California, Keck School of Medicine, 1510 San Pablo Street, Building 4300, Suite #412, Los Angeles, CA 90033, USA

## Abstract

Poorer than expected, living donor liver transplant outcomes are observed after recipient graft artery thrombosis. At grafting, the risk for later thrombosis is high if a dissected hepatic artery is used for standard reconstruction. Surgeon diagnosis of dissection requires nonstandard management with alternative technique in addition to microvascular expertise. Intimal flap repair with standard reconstruction is contingent on basis of a redo anastomosis. It is a suboptimal choice for living donor transplantation. Achieving goal graft arterial perfusion at first revascularization is crucial for superior outcomes. Managing dissection at grafting with nonstandard left gastric artery reconstruction is unreported. Our experience is limited, but this is our preferred alternative technique to standard hepatic artery reconstruction complicated by dissection. Here, we describe our two-case experience with left gastric arterialized grafts for management of dissection. Our living donor graft recipients with alternatively arterialized grafts are now 6- and 2-years posttransplant.

## 1. Introduction

Implantation of the right lobe living donor liver graft begins with vascular reconstruction. The arterial anastomosis is last. Standard hepatic artery reconstruction (HAR) receives inflow from the recipient right, left, or proper hepatic artery (HA) to supply the donor artery. The recipient inflow is inspected for good wall integrity, a similar size, and available length to reach the donor artery for tension free anastomosis [1]. The donor artery determines the diameter and length required. It is anatomically narrow and short, thus with an increased associated risk for injury and hepatic artery thrombosis (HAT) [1–4]. The reported HAT rate ranges from 2% to 6% [5–9]. The lower rate is associated with growing cumulative surgeon microvascular expertise [2, 4–11]. Some say that the lowest HAT rates can be observed after adding an operating microscope to HAR at grafting [1]. Keeping HAT rates low allows for greater and more meaningful utilization of available grafts.

Surgeon expertise and technique are key for a successful living donor liver transplantation. This is especially true at centers with living donor recipient candidates who are sicker at transplant with comorbid conditions that alone increase the estimated risk of HAT at or after grafting. Poor arterial circulation system health condition is revealing of candidacy and increased estimated HAT risk. Living donor graft recipient history of portal vein thrombosis (PVT) or hepatocellular carcinoma (HCC) is reported to increase HAT risk. A history of diagnostic or treatment mesenteric angiography, especially transarterial catheter embolization (TACE) for treatment of HCC, increases risk for HAT, and some authors advise avoiding endovascular procedures months prior to a recipient's scheduled living donor transplant. Studies report partial or occlusive HAT complicating TACE treatment that may take six months for recanalization [12–14]. In addition to these pretransplant conditions, all recipients have slightly increased risk for HAT events during the acute recovery period with a transient hypercoagulable state [2, 4, 10, 15, 16]. Living donor graft recipient HAT and sequela ends with retransplant in 38.1% of those affected. Among recipients without ever having graft HAT, only 3.2% require retransplant after right lobe living donor liver transplantation (RLDLT) [17].

Here, we describe two cases of recipient HA intimal dissection (ID) diagnosed at HAR. Both recipients had ID with progression proximally without propagation to the left gastric artery (LGA). Therefore, the LGA was the most suitable alternate recipient vessel for primary anastomosis and inflow to the graft artery.

## 2. Case 1

A fifty-nine-year-old female with a history of hepatitis C virus (HCV) infection, liver cirrhosis, MELD score of 14, severe portal hypertension, and HCC underwent planned RLDLT. Her HCC history is significant for treatment with TACE using the right posterior HA and the inferior right phrenic artery. She had two prior abdominal surgeries making the recipient operation start more difficult.

**Figure 1:**
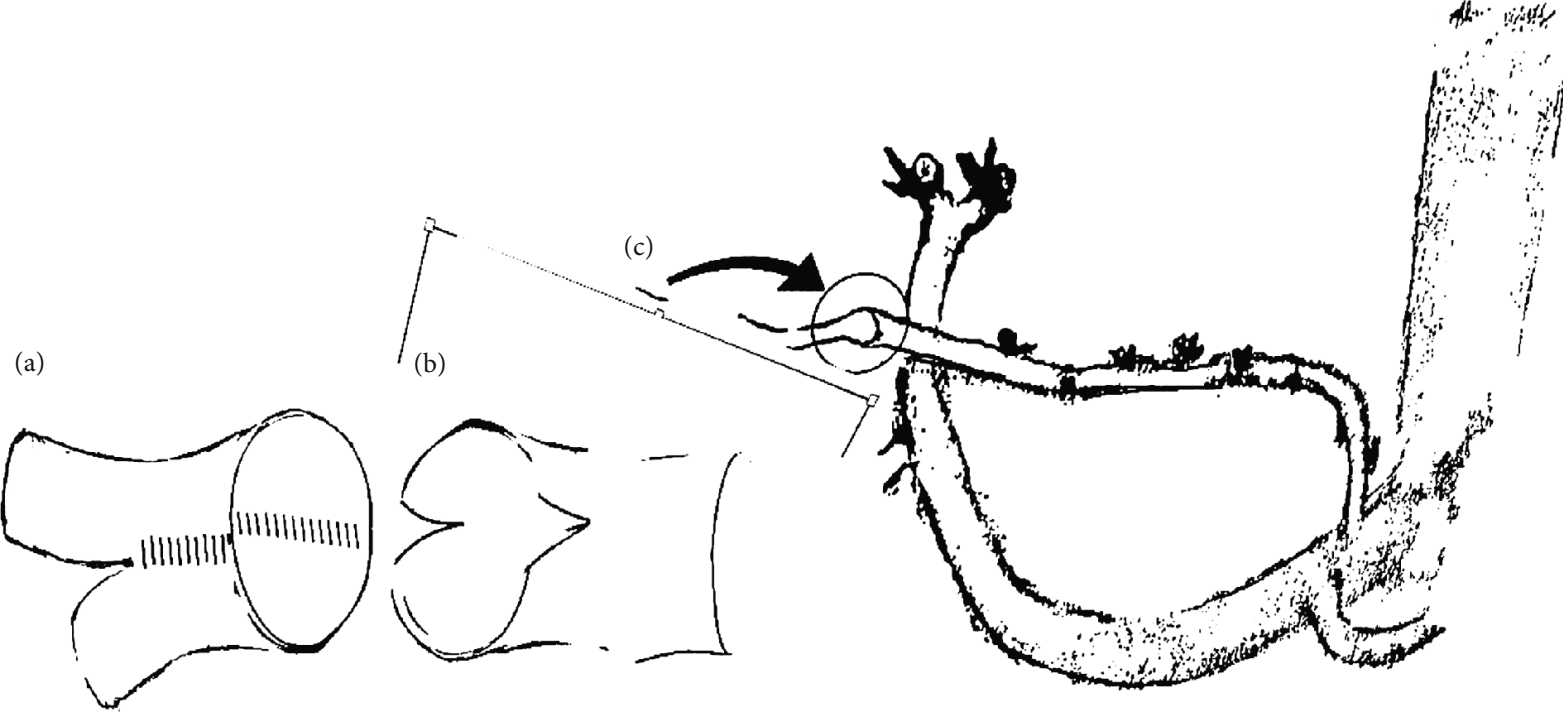
In case 1, the right lobe liver graft has a dual arterial blood supply with a single common orifice for implantation after back-table procedure angioplasty (a). Spatulating the recipient's LGA creates a size-matched inflow for anastomosis with the graft artery. LGA mobilization from the lesser curve proximally to its celiac artery trunk takeoff superficializes the vessel with a natural rightward curve for tension-free anastomosis.

The donor operation was uncomplicated; the right lobe liver graft was delivered. The back table procedure was significant for a dual arterial blood supply and planned angioplasty. The two donor arteries measured 2 to 3 millimeters (mm) in diameter. After spatulating both lumens, a running 8-0 Prolene suture (Figure 1(a)) was used to create the single common orifice for anastomosis in the recipient.

After hepatectomy, the implantation begins with standard vascular reconstruction of the hepatic vein outflow and portal vein inflow. The initial reperfusion is successful, and HAR begins with preparation of the recipient HA. Diagnosis of ID upon intraluminal inspection complicates and precludes final revascularization completion. The severely dissected HA is unusable and ligated. An alternative inflow technique is chosen for management. We free the LGA along the lesser curve for inspection. It has good arterial wall integrity, the lumen is without dissection, the orifice is 2.0 mm wide, and the length is estimated to be adequate for tension free anastomosis. The LGA mobilization then proceeds proximally until its celiac artery trunk takeoff is reached. Vascular shunts, gastric artery branches, and the left gastric vein are carefully controlled with suture ligature. Perigastric shunts are most prominent, requiring more time than usual for vascular control as dissection is carried into the lesser sac. We follow the gastropancreatic fold to the celiac trunk and inferior extent of our mobilization. Surgeon microvascular expertise is required to complete nonstandard reconstruction. The LGA is spatulated to size-match the graft artery common orifice (Figure 1(b)). Primary anastomosis is with interrupted 8–0 Prolene suture.Graft arterial perfusion is restored and graft revascularization in the recipient is completed. The general principle of our strategy for graft artery reconstruction is shown in Figure 1(c). Biliary reconstruction with duct-to-duct anastomosis completes the recipient operation.

### 2.1. Postoperative Course

**Figure 2:**
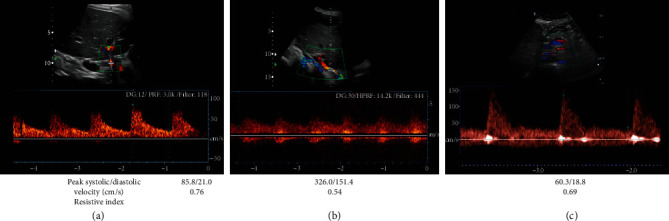
Case 1 baseline Doppler ultrasound with appropriate posttransplant waveforms at the recipient left gastric to graft artery anastomosis (a). Dampened “parvus tardus” waveforms concerning for arterial stenosis at the anastomosis on postoperative day 37 prior to intervention. Appropriate arterial waveforms three-year posttransplant after successful primary balloon angioplasty (c).

On postoperative day (POD) 1, the baseline Doppler ultrasound (US) confirms vascular patency and graft arterial inflow with typical waveforms (Figure 2(a)). However, on POD 37, a repeat US has abnormal “parvus tardus” waveforms at the anastomosis concerning for arterial stenosis (Figure 2(b)). Computed tomography angiography (CTA) and US findings are congruent, and the anastomotic narrowing is successfully treated endovascularly. Three-year postangioplasty US findings (Figure 2(c)) show typical waveforms.

**Figure 3:**
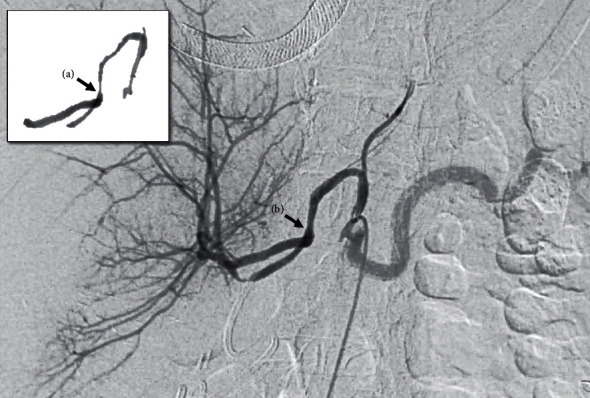
Case 1 celiac angiogram on postoperative day 38. The arrow points to the long segment arterial stenosis preangioplasty (a) and after successful balloon angioplasty (b).

Diagnostic celiac angiography revealed a long segment stenosis at the anastomosis and this was simultaneously treated with balloon angioplasty (Figure 3(a)). The completion angiogram confirms successful initial intervention (Figure 3(b)). No further repeat interventions were necessary. Presently, the patient is alive with a functioning left gastric artery arterialized graft 74 months, or 6 years, after living donor liver transplantation complicated by dissection.

## 3. Case 2

A sixty-four-year-old female with nonalcoholic steatohepatitis (NASH), decompensated liver cirrhosis with a MELD score of 25, portal hypertension, hepatorenal syndrome requiring dialysis, and HCC undergoes RLDLT. The donor operation and back table procedure proceed uneventfully until the graft is ready for implantation.

Grafting in the recipient begins after hepatectomy in similar order as previously described to initial reperfusion. During HAR, the recipient HA is diagnosed with severe ID at intraluminal inspection, andstandard reconstruction is precluded. Following prior initial success, we continue with our preferred alternative strategy for managing the dissection with using the LGA instead. Simply spatulating, the recipient's 2.4 mm LGA matches it to the single 3.2 mm donor artery. Arterial anastomosis is with interrupted 8–0 and 9–0 nylon suture. Initial whole graft arterial perfusion is achieved, and final revascularization is completed. We proceed with biliary reconstruction using Roux-en-Y hepaticojejunostomy creation and end the recipient operation.

### 3.1. Postoperative Course

**Figure 4:**
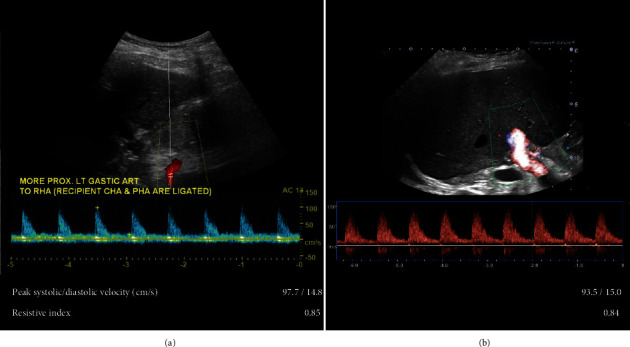
Case 2 Doppler ultrasound (US) imaging on postoperative day 1 with appropriate waveforms at the alternatively reconstructed anastomosis with inflow from the left gastric artery (a). Repeat US nine-month posttransplant with stable findings (b).

On POD 1, the baseline Doppler US confirms vascular patency and appropriate waveforms (Figure 4(a)). Acute recovery and later convalescence are uncomplicated. A repeat US at nine-month posttransplantation is with stable and appropriate findings (Figure 4(b)). Presently, the patient is alive with a functional graft 27 months, or 2 years, after RLDLT.

## 4. Discussion

Living donor graft artery diameter of 2 mm or less is used to exclude donor recipient pairs from RLDLT [4]. Now, accumulated microvascular expertise allows for small donor artery reconstruction [2, 4, 7]. Supporting evidence from published case series shows that cumulative microvascular experience regardless of surgeon specialty reduces HAT rates [4, 7, 8, 10].

Database-driven outcome studies report loss of expected posttransplant benefit in living donor recipients with history of graft artery thrombosis, regardless of redo anastomosis and salvage attempt status. Thus, dissection diagnosed at HAR should prohibit standard revascularization technique. Few report their nonstandard management at grafting, and outcomes are unknown. To our knowledge, this is the first report of observed long-term survival in recipients with left gastric arterialized right liver grafts as the alternative to standard HAR during RLDLT.

RLDLT rates are rising, our technique is better, but graft HAT continues to challenge outcomes after living donation [10, 18]. It is time that we consider and recommend an alternative technique to standard reconstruction with HAT complications and sequela that are irreversible even when salvage is successful. HAR using alternate inflow from the LGA shows achievable long-term success in comparison to standard technique. One study reported 8 out of 113 (7%) living donor recipients with failure of primary arterial anastomosis due to no flow or HAT found at attempted salvage. Salvage attempts may prevent graft loss but do not protect against ischemic injury and sequela, leading to inferior graft outcome.

Currently, described alternative techniques for HAR are done after the ischemic injury has occurred. Redo anastomosis at grafting with alternative inflow from the recipient left or right gastric [19], the right gastroepiploic (RGEA) [19, 20], and the gastroduodenal (GDA) [2] arteries is described.

One author describes graft salvage in a recipient with TACE treatment-related HA injury and successful GDA reconstruction after redo anastomosis [2]. One report using alternative HAR in 12 out of 15 patients describes no lethal complication at almost one-year follow-up after transplant, with 2 using the native right HA, 6 cases with RGEA inflow, 2 instances of redo anastomosis with the native right HA after thrombectomy, 1 case using the left HA, 1 case of the gastroduodenal artery, and 1 with supraceliac inflow [19]. There are 3 out of 15 patients (20%) died from complications, including later biliary sepsis and pneumonia [19].

More distal inflow from the splenic artery or supraceliac aorta requires interposition grafting. Autologous vessel harvest of the sigmoid artery [21], or the inferior mesenteric artery (IMA) [22], and the radial artery are other options. Autologous veins are less favorable. A report on gonadal vein reconstruction is also in the literature, but venous grafts are inferior to arterial ones. Interposition grafting has a poorer outcome versus alternative inflow vessels [4]. Cadaveric vessels, if available, are possible to use with supraceliac anastomosis [4, 19].

Some assess intimal injury with severity grading at grafting to guide intimal repair. Other studies have also reported tacking or trimming surgical techniques for less severe hepatic artery injuries [10]. Agarwal et al. report using the repaired dissected HA for primary anastomosis in 9 patients with moderate to severe ID at transplant. Posttransplant, 1 out of 9 patients developed HAT [10]. In another study, out of 6 patients with HAT, three died from biliary sepsis, graft dysfunction from large-sized ischemic injury, and pneumonia.

The LGA is an attractive option for alternative inflow at reconstruction with a proximal celiac origin. The takeoff from the celiac is hidden from the pathologic cirrhotic environment in vivo, including splanchnic vessel remodeling. The LGA's winding course makes it challenging to access accidentally during scheduled TACE treatments [18]. Its size is an excellent match to the donor artery.

The dissected recipient HA diagnosed at transplant clearly indicates its poor integrity and increased risk for HAT. Our management of ID involves ligating the HA and the immediate use of the LGA for inflow. Our experience with two RL-LDLT recipients supports its suitability and feasibility and, in our opinion, is the best alternate inflow for primary anastomosis. Successful final revascularization, posttransplant graft recipient recovery, and possible long-term survivability are benefits in addition to management dissection at grafting. Both living donor recipients continue to do well with overall survival of greater than 6- and 2-year posttransplant.

## 5. Conclusion

Achievable expected living donor outcomes with left gastric arterialized grafts support using alternative left gastric artery inflow as the best choice for backup to standard HAR complicated by dissection at grafting.

## Data Availability

There are no data to be declared.

## References

[1] Klintmalm G. B. (2015). *Transplantation of the Liver-Expert Consult-Online and Print*.

[2] Okazaki M., Asato H., Takushima A. (2006). Hepatic artery reconstruction with double-needle microsuture in living-donor liver transplantation. *Liver Transplantation*.

[3] Abu-Gazala S., Olthoff K. M. (2019). Current status of living donor liver transplantation in the United States. *Annual Review of Medicine*.

[4] Lin T.-S., Vishnu Prasad N. R., Chen C.-L. (2019). What happened in 133 consecutive hepatic artery reconstruction in liver transplantation in 1 year?. *HepatoBiliary Surgery and Nutrition*.

[5] (2021). Managing recipient hepatic artery intimal dissection during living donor liver transplantation. https://aasldpubs-onlinelibrary-wiley-com.libproxy1.usc.edu/doi/epdf/10.1002/lt.25857.

[6] Olthoff K. M., Smith A. R., Abecassis M. (2015). Defining long-term outcomes with living donor liver transplantation in North America. *Annals of Surgery*.

[7] Uchiyama H., Hashimoto K., Hiroshige S. (2002). Hepatic artery reconstruction in living-donor liver transplantation: a review of its techniques and complications. *Surgery*.

[8] Akbulut S., Kutluturk K., Yilmaz S. (2021). Hepatic artery reconstruction technique in liver transplantation: experience with 3,000 cases. *Hepatobiliary Surgery and Nutrition*.

[9] Herrero A., Souche R., Joly E. (2017). Early hepatic artery thrombosis after liver transplantation: what is the impact of the arterial reconstruction type?. *World Journal of Surgery*.

[10] Agarwal S., Dey R., Pandey Y., Verma S., Gupta S. (2020). Managing recipient hepatic artery intimal dissection during living donor liver transplantation. *Liver Transplantation*.

[11] Uchiyama H., Shirabe K., Taketomi A. (2010). Extra-anatomical hepatic artery reconstruction in living donor liver transplantation: can this procedure save hepatic grafts?. *Liver Transplantation*.

[12] Goel A., Mehta N., Guy J. (2014). Hepatic artery and biliary complications in liver transplant recipients undergoing pretransplant transarterial chemoembolization. *Liver Transplantation*.

[13] Kulik L., Heimbach J. K., Zaiem F. (2018). Therapies for patients with hepatocellular carcinoma awaiting liver transplantation: a systematic review and meta-analysis.

[14] Onizuka H., Sueyoshi E., Ishimaru H., Sakamoto I., Uetani M. (2017). Arterial injury during transcatheter arterial chemoembolization for hepatocellular carcinoma: predictors of risk and outcome. *Abdominal Radiology*.

[15] (2022). Prevention of hepatic artery thrombosis in pediatric liver transplantation. https://oce-ovid-com.libproxy2.usc.edu/article/00007890-199511270-00009/PDF.

[16] Ince V., Ersan V., Karakas S. (2017). Does preoperative transarterial chemoembolization for hepatocellular carcinoma increase the incidence of hepatic artery thrombosis after living-donor liver transplant?. *Experimental and Clinical Transplantation*.

[17] Braun H. J., Grab J. D., Dodge J. L. (2021). Retransplantation after living donor liver transplantation: data from the adult to adult living donor liver transplantation study. *Transplantation*.

[18] Lautt W. W. (2009). Hepatic circulation: physiology and pathophysiology. *Colloquium Series on Integrated Systems Physiology: From Molecule to Function*.

[19] Park G.-C., Moon D.-B., Kang S.-H. (2019). Overcoming Hepatic Artery Thrombosis after Living Donor Liver Transplantations: An Experience from Asan Medical Center. *Annals of Transplantation*.

[20] Wang C.-C., Lin T.-S., Chen C.-L., Concejero A. M., Iyer S. G., Chiang Y. C. (2008). Arterial reconstruction in hepatic artery occlusions in adult living donor liver transplantation using gastric vessels.

[21] Inomoto T., Nishizawa F., Terajima H. (1995). The use of the recipient sigmoid artery for a revision of hepatic arterial reconstruction after thrombosis in living related liver transplantation. *Transplantation*.

[22] Asonuma K., Ohshiro H., Izaki T. (2004). Rescue for rare complications of the hepatic artery in living donor liver transplantation using grafts of autologous inferior mesenteric artery. *Transplant International*.

